# Spatio-temporal patterns of neurodegenerative disease hospitalizations in mainland Portugal

**DOI:** 10.3389/fpubh.2026.1767007

**Published:** 2026-06-04

**Authors:** Mariana Oliveira, Alberto Freitas, Ana Cláudia Teodoro, Hernâni Gonçalves

**Affiliations:** 1 RISE-Health, Department of Community Medicine, Information and Health Decision Sciences, Faculty of Medicine, Porto, Portugal; 2 Department of Geosciences, Environment and Land Planning, Faculty of Sciences, University of Porto, Porto, Portugal; 3 Earth Sciences Institute (ICT), Pole of the FCUP, University of Porto, Porto, Portugal

**Keywords:** environmental health, hospitalization rates, neurodegenerative diseases, spatial epidemiology, spatio-temporal analysis

## Abstract

**Background:**

Neurodegenerative diseases are an increasing concern for the aging population worldwide. In Portugal, as in many other developed countries, the population is aging rapidly. Understanding temporal and spatial patterns is of utmost relevance to help manage the burden these diseases place on the healthcare system.

**Methods:**

In this retrospective study, we analyzed over 500,000 hospitalizations discharged between 2000 and 2016. We used the empirical Bayes method to compute the smoothed age-standardized hospitalization rates for each neurodegenerative disease, for all hospitalizations per year, and across administrative divisions (districts and municipalities). We then searched for data clusters using both global and local spatial autocorrelation methods based on the Moran index.

**Results:**

A steady increase in age-standardized hospitalization rates was observed throughout the study period. Statistically significant global spatial autocorrelation was found when considering all diseases per municipality (Moran’s *I* = 0.010, *p*-value < 0.001). In addition, when considering the districts, only Alzheimer’s disease, dementia, and basal ganglia disorders did not show significant spatial autocorrelation. When considering municipalities, all diseases showed significant positive global spatial autocorrelation.

**Conclusion:**

Temporal analysis showed increasing age-standardized hospitalization rates over time, likely reflecting the aging population in Portugal. The spatial analysis showed significant clustering, which may reflect geographic differences in hospitalization practices, access to care, population structure, or other contextual factors. Through this study, we hope to enlighten future research by providing insights into the anticipated spatio-temporal patterns.

## 1 Introduction

The increase in global life expectancy from 66.8 to 73.3 years between 2000 and 2019 (1) has increased concerns about neurodegenerative diseases, since age is one of the primary risk factors for developing these diseases that occur due to pathological changes in the brain (2) and result in the death of cells due to either necrosis or delayed apoptosis (3).

Alzheimer’s disease (AD) and other forms of dementia are the most common types of late-onset dementia globally, contributing to 12% of the burden in disability-adjusted life years (4). Along with Parkinson’s disease (PD), these are the most prevalent neurodegenerative diseases worldwide (5, 6). Several complications, such as longer hospital stays, higher mortality rates, and an increased likelihood of being discharged to long-term care, are experienced by these patients (7). Other neurodegenerative diseases include, but are not limited to, multiple sclerosis (MS), amyotrophic lateral sclerosis (ALS), and Huntington’s disease.

Some risk factors for these diseases have been discussed, including age, sex, genetic susceptibility, and environmental exposure to pollution. For example, one-third of Alzheimer’s disease patients are women, whereas Parkinson’s disease is more prevalent in men (5). Huntington’s disease is entirely hereditary, and some forms of early-onset Alzheimer’s disease are also genetically transmissible (8). In addition to individual-level risk factors, regional differences in environmental exposure, socioeconomic conditions, and access to healthcare may contribute to geographic variability in neurodegenerative disease burden.

Furthermore, the importance of spatially analyzing neurodegenerative diseases and their relevance to uncover new correlations with variables, such as air pollution and latitude, has been emphasized in several studies (9). These factors can vary geographically, including within Portugal, which exhibits marked regional heterogeneity in demographic structure, urbanization, and healthcare access (10). Effective planning and mitigation of the burden posed by these disorders depends heavily on the spatial representation and understanding of the characteristics and living environment of patients.

Although previous studies have described the epidemiology and risk factors of individual neurodegenerative diseases, there is still limited evidence of their long-term geographic distribution at the national level, particularly across multiple diseases and spatial scales (11). In Portugal, this gap is relevant because regional differences in aging, urbanization, healthcare access, and environmental exposure may influence hospitalization patterns (12). This retrospective study uses secondary hospitalization data from 2000 to 2016 to provide a nationwide spatio-temporal characterization of neurodegenerative disease hospitalizations in mainland Portugal at both the district and municipality levels, offering a descriptive basis for future studies exploring environmental, socioeconomic, and healthcare-related determinants.

## 2 Materials and methods

### 2.1 Study area

**Figure 1:**
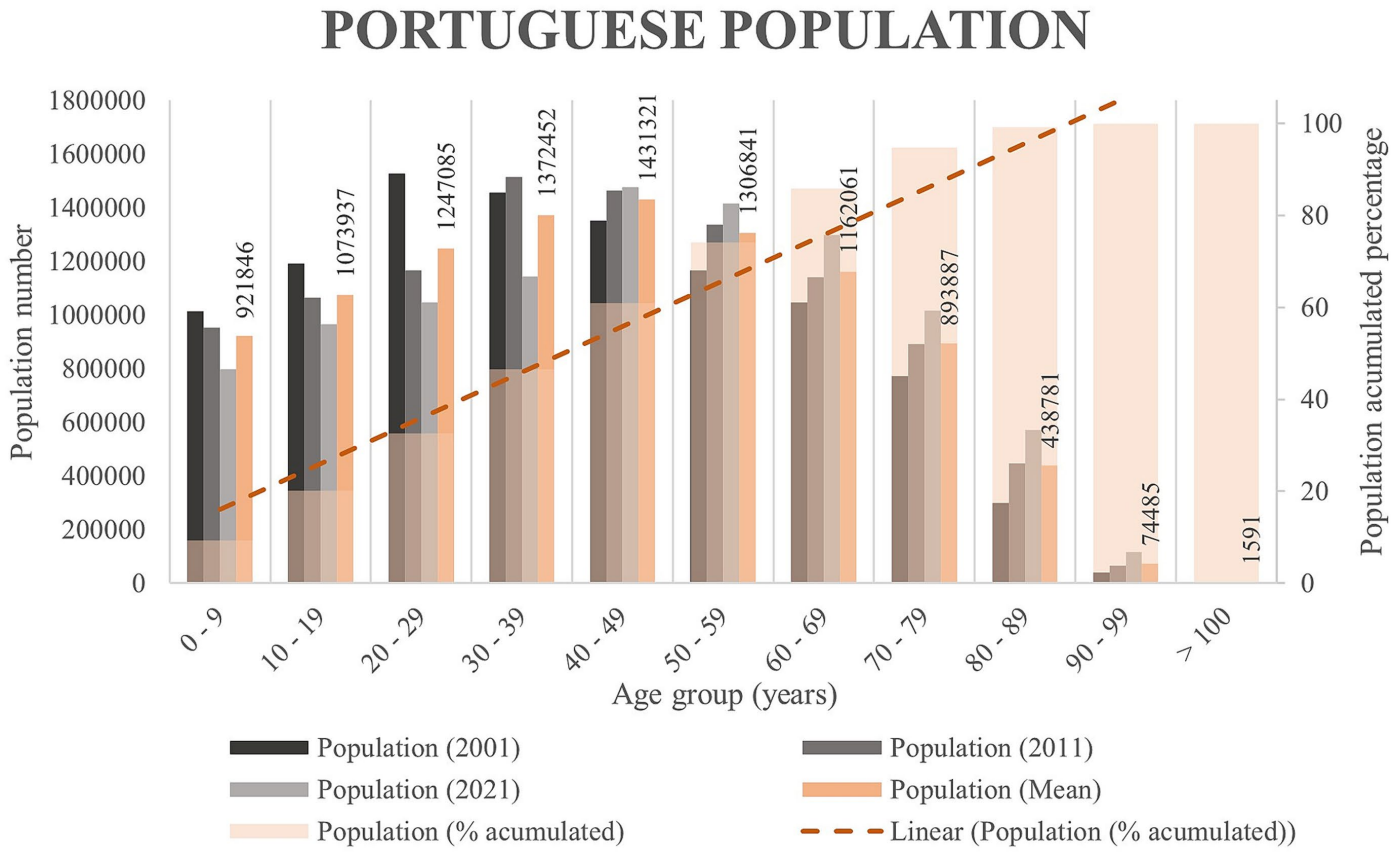
Population distribution per age group in Portugal: mean value from 2001 to 2021 [10].

This study was conducted using data obtained from mainland Portugal, a country located in the southwest of Europe. It has an area of approximately 89.000 km^2^ and a population of nearly10,000,000 inhabitants, with a highly heterogeneous age distribution (Figure 1). Of this population, 21% of are 65 years old or older (13).

**Figure 2:**
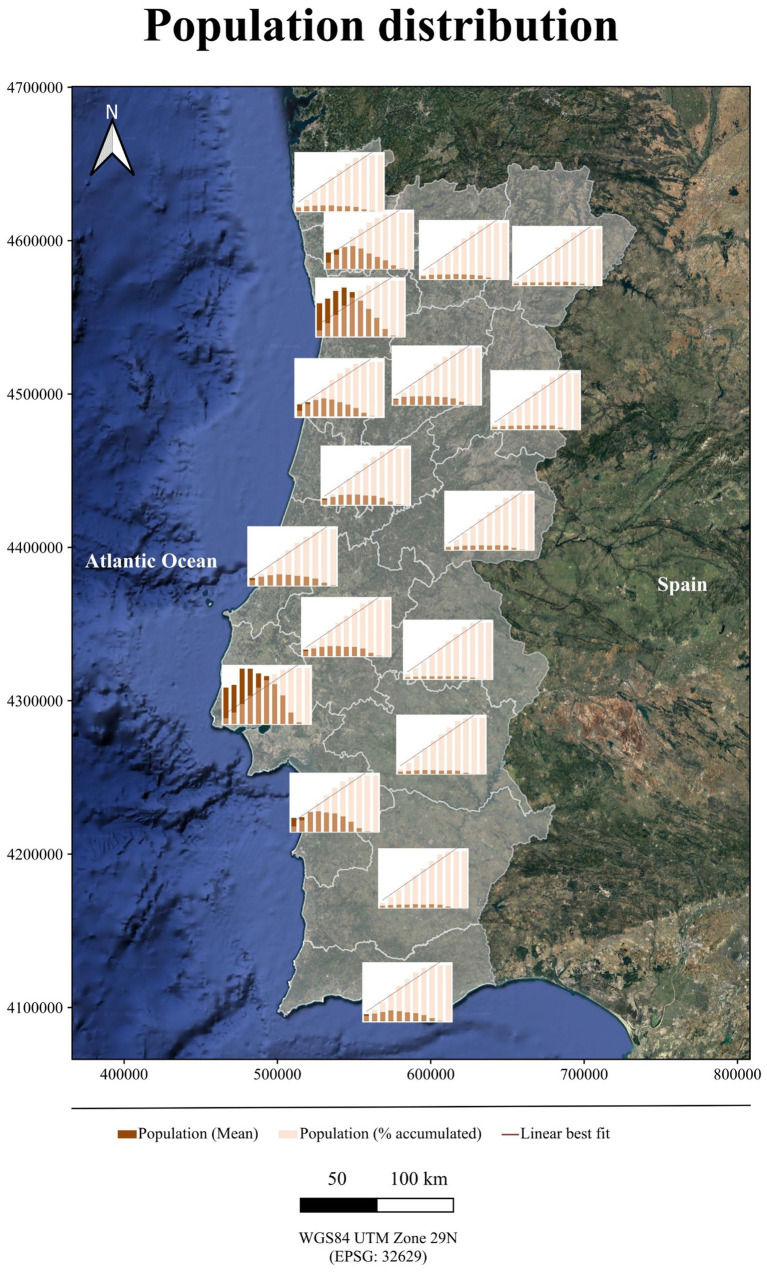
Country’s population distribution per district and age group—population size = [0–350,000], population % accumulated = [0–100], and age groups as in Figure 1. Base map from Google Earth, Map data © 2026 Google. Population date from “INE - Population data” National Institute of Statistics, IP – Portugal, licensed under CC BY 4.0. Municipalities / districts data from “DGT - Administrative Limits”, General Directorate of Territory.

The most populated cities in Portugal are Porto and Lisbon, as shown in the map in Figure 2, and the interior of the country shows a very low population density. Portugal is administratively divided into districts and municipalities, which constitute the two spatial scales used in this study. Districts represent larger regional administrative units, whereas municipalities are smaller local administrative areas, allowing for analysis at both broader and finer spatial resolutions.

### 2.2 Data acquisition

#### 2.2.1 Health data

Hospitalizations of patients with at least one neurodegenerative pathology coded as a diagnosis, either primary or secondary, were obtained from secondary hospitalization data from public hospitals with discharge dates between 2000 and 2016. No further years were included because the provided dataset included only hospitalizations from 2000 to 2018; however, both 2017 and 2018 were incomplete and would thus result in non-comparable results. Each record contained the year, patient’s sex, age, residence (coded to the parish), admission and discharge dates, length of stay, and diagnosis codes (ICD-9-CM or ICD-10-CM codes).

International Classification of Diseases (ICD) codes for neurodegenerative pathologies were retrieved from a literature review (14–19), in consultation with coding experts, and are listed in Table 1. The category “Other” represents neurodegenerative diseases not described elsewhere by the alternative codes and is a category included in the ICD.

**Table 1:** ICD codes used to retrieve hospitalization data.

Diagnosis		ICD-9	ICD-10
Alzheimer’s		331.0	G30
Pick’s		331.1	G31
Lewy		331.8	
Creutzfeldt		046	A81.0
Huntington’s		333.4	G10
Parkinson’s		332	G20
Motor neuron		335	G12.2
Multiple sclerosis		340	G35
Basal ganglia disorders		333.0	G23
Dementia	Vascular	290.4	F01 F02 F03
	Unspecified	294	
	Senile	331.2	
	Cerebral degeneration	331.7	
	Presenile	290.1	
	Senile with delusion	290.2	
	Senile with delirium	290.3	
	Alcohol induced	291.2	
	Drug induced	292.82	
Other		331.9	G32

#### 2.2.2 Population data

Population data for each Portuguese mainland parish were retrieved from the National Institute of Statistics (INE) publicly available Population Census for 2001, 2011, and 2021. To obtain an approximate value of the population by year, data were then linearly interpolated and used to normalize the health data. All values were rounded to the nearest integer owing to the nature of the population variable. The district and municipality populations were calculated by summing the parishes.

### 2.3 Data processing

*Python* was used in all stages of data preprocessing, descriptive analysis, and mapping.

Diagnosis codes were extracted from the health dataset and recoded according to the corresponding disease category (for example, ICD-9 code 331.0 was recoded as “Alzheimer’s disease,” as well as ICD-10 code G30). Age was grouped into three categories: under 20 years, 20 to 70 years, and over 70 years. These groups were chosen because they distinguish pediatric (20), early-onset, and late-onset neurodegenerative disease (21) hospitalizations and because they matched the population denominator data consistently available across the study period and spatial units. Using broader age groups also helped reduce sparse counts in smaller municipalities.

The data were obtained by parishes but were also grouped by district and municipality. The population in each administrative area and the number of patients per age group were used to compute the age-standardized hospitalization rates (ASHR) using Equation 1 (22). ASHR is a form of normalizing data, therefore enabling the comparison of rates across space and time with a lower degree of bias.

$$\text{ASHR}=\frac{N_{\text{AgeGroup}}}{\text{Populatio}n_{\text{AgeGroup}}}\times {\%}_{\text{AgeGroup}}$$ (1)

In Equation 1, *N_AgeGroup_* is the number of hospitalizations per age group, *Population_AgeGroup_* is the total population in the administrative area for each age group, and *%_AgeGroup_* is the percentage that the age group represents in the overall population of that administrative area.

Given the very low population in some municipalities, an empirical Bayesian approach was used to smooth the ASHR [19] and reduce the statistical instability caused by the problem of small numbers [20]. This approach consisted of creating a distance-based weights matrix for each administrative area, which assigned a weight of 0 to areas beyond the distance threshold and a non-linear weight from 0 to 1 to the closest area. A fixed distance band of 150 km was used to ensure spatial connectivity across mainland Portugal and to provide each administrative area with a meaningful set of neighbors for smoothing and spatial autocorrelation analyses. Because this is a methodological choice, alternative specifications, such as different distance thresholds or contiguity-based matrices, can produce variations in the detected clustering patterns.

Subsequently, global and local spatial autocorrelations were computed to identify the presence of clustering using Moran’s *I* (23) and Local Indicators of Spatial Association (LISA) using the *Python* package *esda*, which implements these statistics directly (24). Both methods assess how likely it is to obtain a map like the one observed due to a random pattern, and if there is no evidence of randomness, then it is assumed there is a statistically significant spatial autocorrelation (25).

## 3 Results

### 3.1 Descriptive statistics

**Figure 3:**
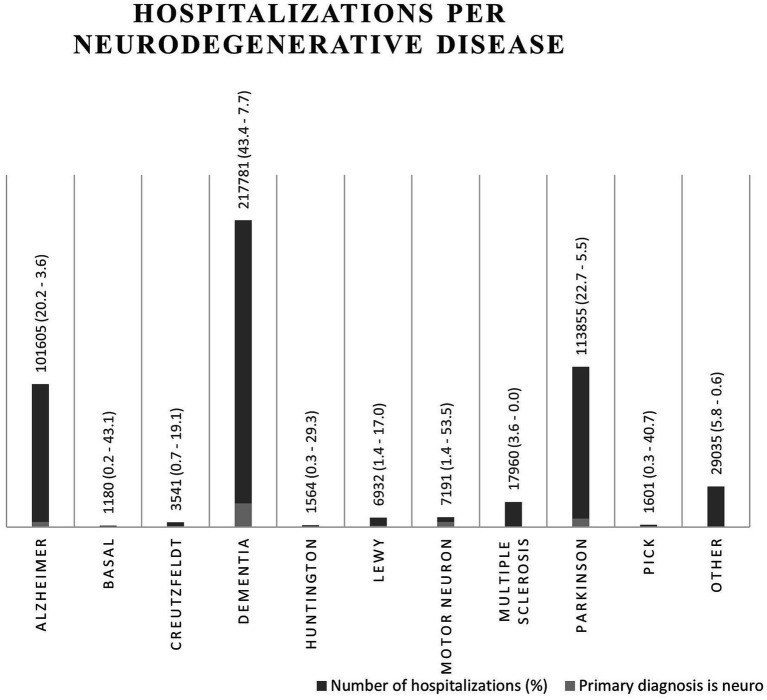
Number of hospitalizations and respective percentage per neurodegenerative disease, followed by the percentage of hospitalizations in which the neurodegenerative disease was a primary diagnosis (the total number of hospitalizations is 502,245).

In this study, 502,245 hospitalizations were included, 51,220 of which presented with two or more neurodegenerative diseases, and 34,250 hospitalizations with a neurodegenerative disease as the primary diagnosis. As shown in Figure 3, the most prevalent disease was Dementia, with 43.4% of the cases, followed by Parkinson’s disease (22.7%) and Alzheimer’s disease (20.2%). The least prevalent disease was dementia (43.4%), with cases of basal ganglia disorders, with 0.2% of cases, Pick’s disease (0.3%), and Huntington’s disease (0.3%). Hospitalizations for motor neuron disease were most often coded as the primary diagnosis (53.5%), followed by basal ganglia disorders and Pick’s disease, which also showed high proportions (43.1 and 40.7%, respectively).

Most patients were female (56.3%) or aged over 70 years (81.3%), and only 0.4% of the cases were of patients under 20 years of age, with motor neuron disease (MND) being by far the most prevalent pediatric neurodegenerative disorder (Table 2).

**Table 2:** Descriptive statistics of each neurodegenerative disease included in this study, as well as the total.

		Alzheimer’s	Basal	Creutzfeldt	Dementia	Huntington’s	Lewy	Motor neuron	Multiple Sclerosis	Parkinson’s	Pick’s	Other	Total
Sex	Female	62,349 (61.4)	659 (55.8)	1,209 (34.1)	127,694 (58.6)	736 (47.1)	3,458 (49.9)	3,272 (45.5)	12,062 (67.2)	56,977 (50.0)	722 (45.1)	13,775 (47.4)	282,913 (56.3)
	Male	39,256 (38.6)	521 (44.2)	2,332 (65.9)	90,087 (41.4)	828 (52.9)	3,474 (50.1)	3,919 (54.5)	5,898 (32.8)	56,877 (50.0)	879 (54.9)	15,260 (52.6)	219,331 (43.7)
Age (years)		81 [76, 85]	72 [61, 79]	48 [38, 68]	82 [76, 87]	58 [47, 70]	78 [70, 84]	65 [53, 73]	43 [33, 54]	79 [73, 84]	68 [60, 77]	76 [66, 83]	80 [74, 86]
Age group	<20 years	16 (0.0)	39 (3.3)	116 (3.3)	157 (0.1)	29 (1.9)	130 (1.9)	748 (10.4)	403 (2.2)	59 (0.1)	2 (0.1)	187 (0.6)	1886 (0.4)
	20–70 years	9,018 (8.9)	511 (43.3)	2,649 (74.8)	25,241 (11.6)	1,169 (74.7)	1,656 (23.9)	4,087 (56.8)	16,775 (93.4)	20,052 (17.6)	956 (59.7)	9,740 (33.5)	91,854 (18.3)
	>70 years	92,571 (91.1)	630 (53.4)	776 (21.9)	192,383 (88.3)	366 (23.4)	5,146 (74.2)	2,356 (32.8)	782 (4.4)	93,744 (82.3)	643 (40.2)	19,108 (35.8)	408,505 (81.3)
Length of stay (days)		8 [4, 13]	8 [4, 15]	15 [7, 34]	8 [5, 14]	9 [4, 16]	9 [5, 16]	7 [3, 13]	5 [2, 9]	8 [4, 14]	12 [6, 24]	9 [5, 17]	8 [4, 14]
Residence area	North	37,116 (36.5)	513 (43.5)	973 (27.5)	79,156 (36.3)	437 (27.9)	2,687 (38.8)	2,396 (33.3)	5,411 (30.1)	35,190 (30.9)	554 (34.6)	22,274 (76.7)	186,707 (37.2)
	Center	49,750 (49.0)	518 (43.9)	1897 (53.6)	114,337 (52.5)	882 (56.4)	3,642 (52.5)	3,621 (50.4)	9,487 (52.8)	61,947 (54.4)	901 (56.3)	5,225 (18.0)	252,207 (50.2)
	South	14,739 (14.5)	149 (12.6)	671 (18.9)	24,288 (11.2)	245 (15.7)	603 (8.7)	1,174 (16.3)	3,062 (17.0)	16,718 (14.7)	146 (9.1)	1,536 (5.3)	63,331 (12.6)
Total		101,605 (20.2)	1,180 (0.2)	3,541 (0.7)	217,781 (43.4)	1,564 (0.3)	6,932 (1.4)	7,191 (1.4)	17,960 (3.6)	113,855 (22.7)	1,601 (0.3)	29,035 (5.8)	502,245 (100)

Categorical data (sex, age group, and residence) are described by absolute frequency and percentage, while continuous data (age and length of stay, which are asymmetrically distributed) are described by their median and interquartile range. The total row describes the frequency of each disease and its percentage.

As for patients with multiple neurodegenerative diseases, most had 2 neurodegenerative diagnoses upon hospitalization (48,692—95.1%), with only 45 having four neurodegenerative diagnoses (0.1%). The most common combination of neurodegenerative diseases was Alzheimer’s disease and dementia (37.3%), with a median age of 82 years (IQR: 77, 86), followed by Parkinson’s disease and dementia (28.2%), with a median age of 81 years (IQR: 76, 86) (Supplementary Table S1).

In addition, patients with neurodegenerative diseases as the primary diagnosis upon hospitalization are summarized in Supplementary Table S2. Dementia remains the most prevalent primary diagnosis, with 49.2% of cases, while Multiple Sclerosis has no cases coded as a primary hospitalization diagnosis.

### 3.2 Spatio-temporal analysis

**Figure 4:**
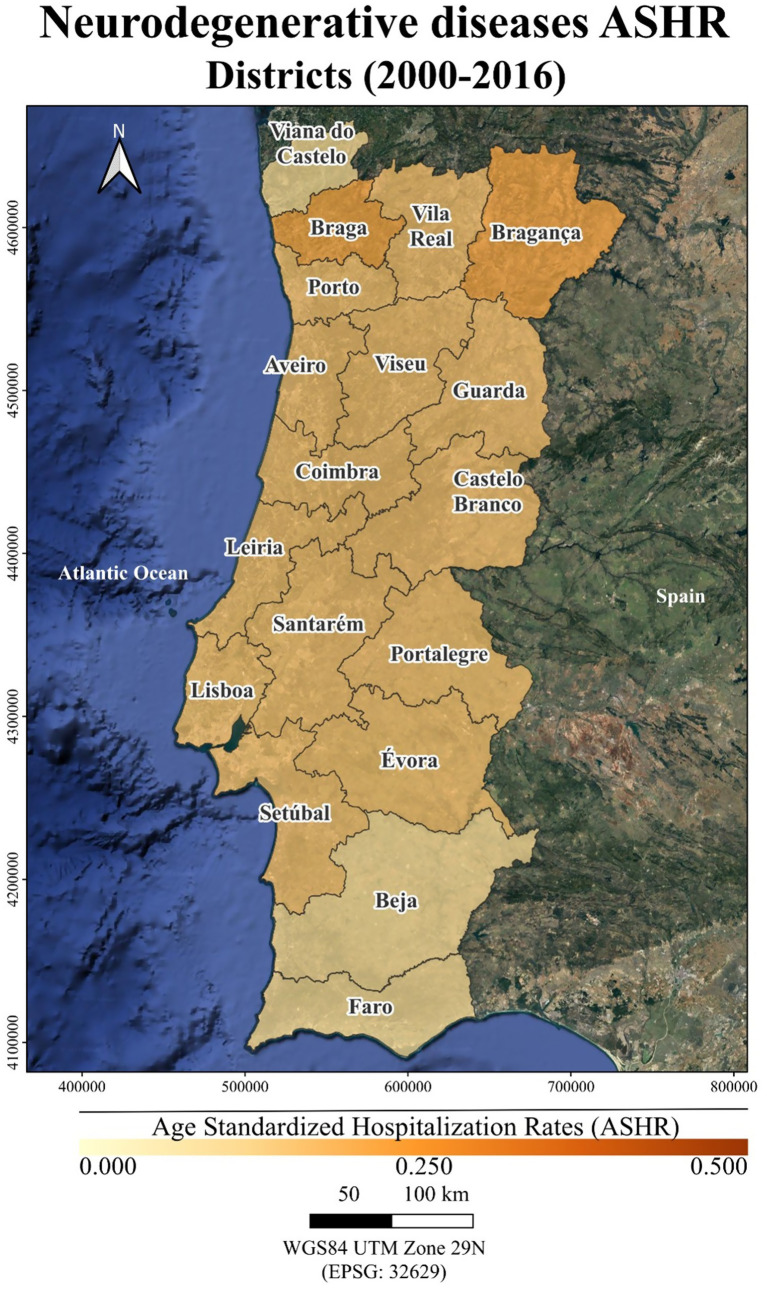
Age-standardized hospitalization rates considering all diseases per district. Base map from Google Earth, Map data © 2026 Google. Municipalities / districts data from “DGT - Administrative Limits”, General Directorate of Territory.

After empirical Bayes smoothing, these rates were lower overall, with Beja (0.119) and Viana do Castelo (0.102) having the lowest rates, whereas Braga (0.207) and Bragança (0.203) had the highest rates (Figure 4). The global average ASHR was 0.168.

#### 3.2.1 Temporal analysis

Table 3 shows the age-standardized hospitalization rates for each district by year. The colors used were the same as those used in the color ramp for mapping the ASHR.

**Table 3:** ASHR per year and per district.

DI	2000	2001	2002	2003	2004	2005	2006	2007	2008	2009	2010	2011	2012	2013	2014	2015	2016
Aveiro	0.040	0.049	0.054	0.066	0.074	0.086	0.098	0.111	0.135	0.156	0.184	0.225	0.257	0.301	0.332	0.348	0.329
Beja	0.026	0.031	0.039	0.046	0.046	0.059	0.066	0.069	0.095	0.109	0.135	0.156	0.183	0.215	0.221	0.271	0.262
Braga	0.038	0.044	0.054	0.071	0.080	0.097	0.116	0.133	0.170	0.192	0.222	0.263	0.326	0.379	0.427	0.463	0.441
Bragança	0.055	0.060	0.064	0.070	0.083	0.101	0.116	0.128	0.172	0.185	0.208	0.251	0.301	0.365	0.395	0.450	0.455
Castelo Branco	0.049	0.054	0.059	0.065	0.082	0.092	0.100	0.110	0.134	0.154	0.195	0.245	0.260	0.304	0.327	0.376	0.378
Coimbra	0.048	0.055	0.057	0.068	0.081	0.093	0.103	0.116	0.141	0.155	0.180	0.217	0.237	0.282	0.299	0.338	0.352
Évora	0.038	0.039	0.048	0.054	0.064	0.077	0.086	0.093	0.119	0.142	0.178	0.216	0.245	0.286	0.303	0.365	0.345
Faro	0.029	0.031	0.040	0.041	0.044	0.052	0.060	0.069	0.094	0.124	0.145	0.156	0.256	0.297	0.282	0.389	0.366
Guarda	0.051	0.060	0.065	0.076	0.082	0.101	0.113	0.129	0.166	0.194	0.203	0.225	0.261	0.317	0.324	0.343	0.351
Leiria	0.048	0.054	0.058	0.072	0.086	0.101	0.114	0.129	0.153	0.171	0.217	0.260	0.294	0.346	0.363	0.391	0.419
Lisboa	0.046	0.052	0.059	0.069	0.084	0.100	0.111	0.128	0.151	0.168	0.211	0.248	0.266	0.323	0.354	0.392	0.452
Portalegre	0.041	0.044	0.047	0.060	0.069	0.085	0.097	0.104	0.132	0.150	0.179	0.216	0.233	0.286	0.296	0.344	0.354
Porto	0.037	0.046	0.053	0.064	0.072	0.089	0.104	0.117	0.150	0.168	0.183	0.217	0.256	0.295	0.327	0.365	0.368
Santarém	0.043	0.048	0.054	0.067	0.076	0.092	0.101	0.111	0.139	0.163	0.200	0.249	0.271	0.309	0.320	0.371	0.364
Setúbal	0.032	0.035	0.042	0.049	0.054	0.066	0.076	0.083	0.114	0.136	0.165	0.194	0.239	0.289	0.302	0.376	0.370
Viana do Castelo	0.016	0.021	0.028	0.035	0.042	0.043	0.051	0.062	0.078	0.088	0.099	0.115	0.141	0.184	0.216	0.259	0.257
Vila Real	0.037	0.047	0.056	0.065	0.069	0.087	0.098	0.117	0.152	0.177	0.183	0.206	0.243	0.303	0.327	0.345	0.318
Viseu	0.046	0.055	0.061	0.070	0.080	0.097	0.111	0.127	0.164	0.186	0.205	0.238	0.282	0.325	0.343	0.387	0.373

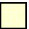
 <0.05, 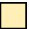 0.05–0.10, 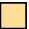 0.10–0.15, 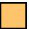 0.15–0.20, 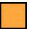 0.20–0.25, 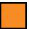 0.25–0.30, 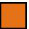 0.30–0.35, 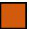 0.35–0.40, 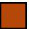 0.40–0.45, 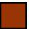 >0.45.

**Figure 5:**
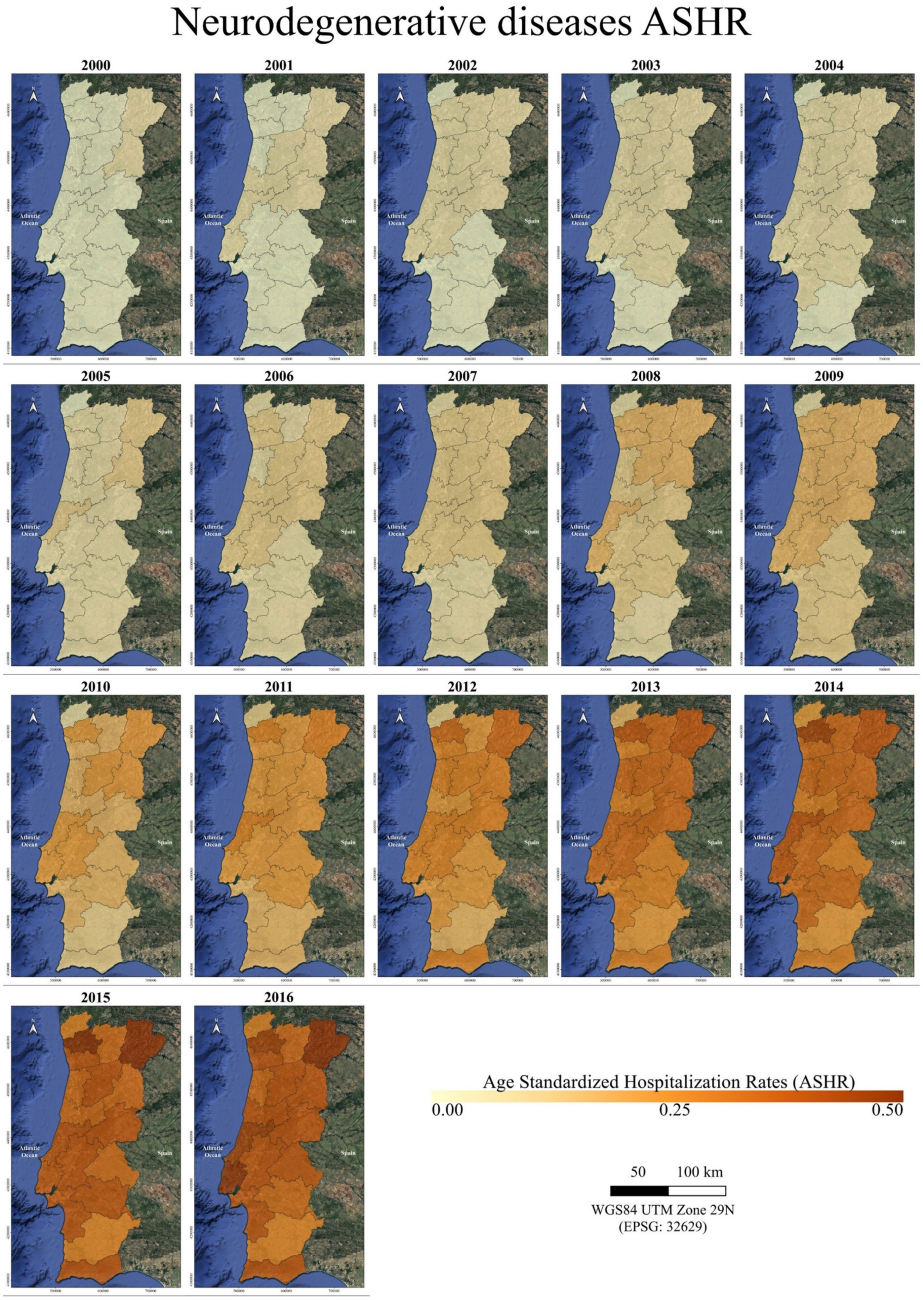
Age-standardized hospitalization rates considering all diseases per district and per year. Base map from Google Earth, Map data © 2026 Google. Municipalities / districts data from “DGT - Administrative Limits”, General Directorate of Territory.

Mapping each year’s age-standardized hospitalization smoothed rates (Figure 5) showed that these rates have been steadily increasing in the past years for most districts. The Braga, Évora, and Faro districts did not seem to have as much increase in hospitalization rates as other districts.

#### 3.2.2 Spatial autocorrelation

Spatial autocorrelation has been studied using both global and local methods. Table 4 shows the results of the global Moran’s autocorrelation index. Focusing on the districts, in general, there was no statistically significant spatial autocorrelation (Moran’s *I* = 0.001, *p*-value = 0.456), even though only Alzheimer’s disease and dementia did not show any statistically significant spatial autocorrelation. When studying municipalities, all values became significant, with global autocorrelation being higher than before (0.005). All diseases showed statistically significant spatial autocorrelation, with the most prominent being the other category, with a Moran’s *I* of 0.061.

**Table 4:** Global Moran’s Index (*p*-value) for each administrative division type studied, both considering all diagnoses and primarily diagnosed neurodegenerative diseases, and each disease.

		All	Alzheimer’s	Basal	Creutzfeldt	Dementia	Huntington’s	Lewy	Motor neuron	Multiple Sclerosis	Parkinson’s	Pick’s	Other
Districts	All diagnoses	0.001 (0.456)	0.000 (0.719)	−0.013 (<0.001)	0.034 (<0.001)	0.003 (0.085)	0.016 (<0.001)	0.018 (<0.001)	0.007 (<0.001)	0.013 (<0.001)	0.005 (0.011)	0.026 (<0.001)	0.082 (<0.001)
	Primary diagnoses	0.025 (<0.001)	0.037 (<0.001)	0.039 (<0.001)	0.011 (<0.001)	0.010 (<0.001)	0.030 (<0.001)	0.029 (<0.001)	0.018 (<0.001)	–	0.022 (<0.001)	0.023 (<0.001)	0.000 (0.729)
Municipalities	All diagnoses	0.005 (<0.001)	0.005 (<0.001)	0.012 (<0.001)	0.013 (<0.001)	0.008 (<0.001)	0.009 (<0.001)	0.014 (<0.001)	0.006 (<0.001)	0.018 (<0.001)	0.013 (<0.001)	0.017 (<0.001)	0.061 (<0.001)
	Primary diagnoses	0.027 (<0.001)	0.019 (<0.001)	0.016 (<0.001)	0.004 (<0.001)	0.015 (<0.001)	0.009 (<0.001)	0.012 (<0.001)	0.009 (<0.001)	–	0.014 (<0.001)	0.011 (<0.001)	0.005 (<0.001)

When considering only patients whose primary diagnosis was neurodegenerative disease, most indices increased, except for Creutzfeldt–Jakob disease, Pick’s disease, and other categories. This category of disease is also the only one in which the significance of the index decreased and was no longer significant when examining the districts’ administrative areas. Furthermore, the most prominent spatial autocorrelation in this case was basal ganglia disorders when considering the districts (0.037) and Alzheimer’s disease when considering the municipalities (0.019).

##### 3.2.2.1 Districts analysis

**Figure 6:**
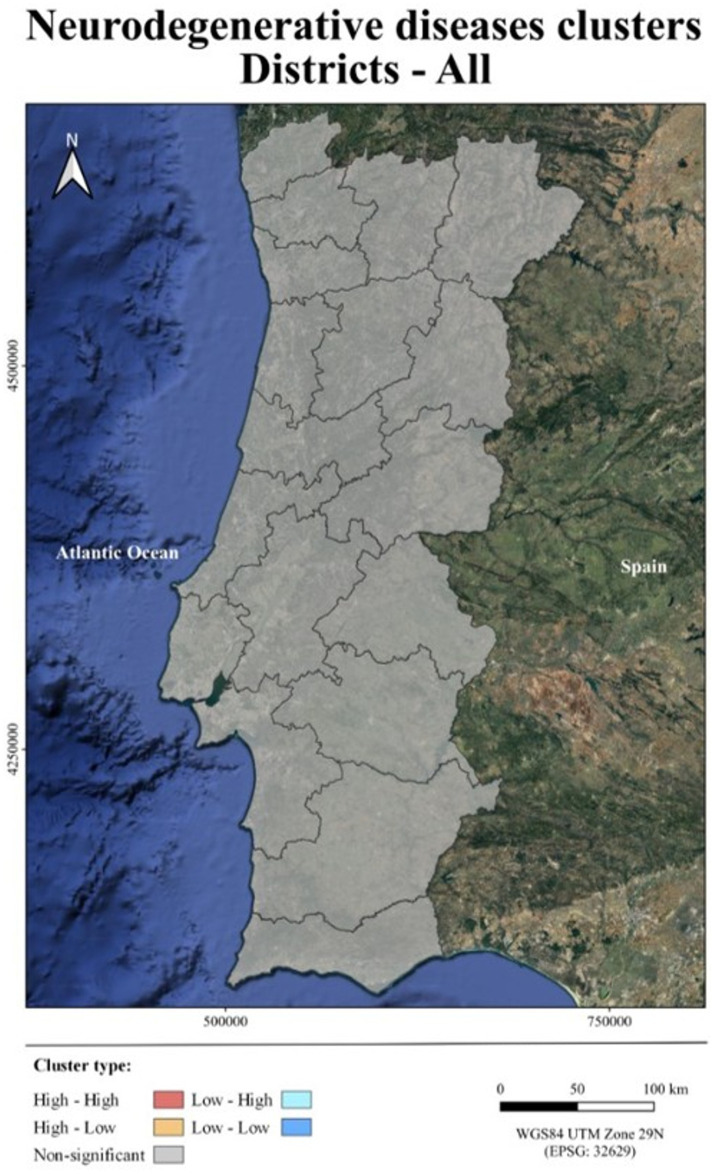
Local neurodegenerative disease clusters when considering districts as administrative areas (all diseases). Base map from Google Earth, Map data © 2026 Google. Municipalities / districts data from “DGT - Administrative Limits”, General Directorate of Territory.

No statistically significant global autocorrelation was found when considering the districts for all diseases, meaning that the degree of clustering could be expected if the samples were random. In addition, when plotting the local spatial autocorrelation (Figure 6), no clusters were found.

**Figure 7:**
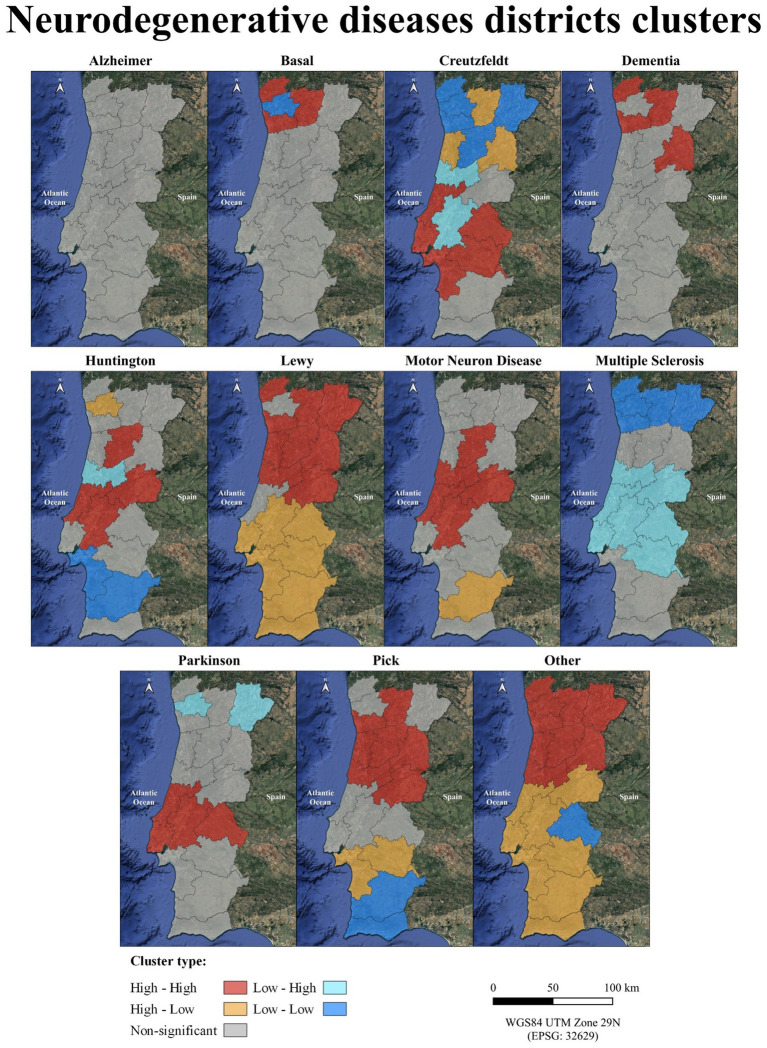
Local neurodegenerative disease clusters when considering districts as administrative areas (by disease). Base map from Google Earth, Map data © 2026 Google. Municipalities / districts data from “DGT - Administrative Limits”, General Directorate of Territory.

When examining each disease individually, neither Alzheimer’s disease nor dementia showed significant global spatial autocorrelation, contrary to basal ganglia disorders, Creutzfeldt–Jakob disease, Huntington’s disease, Lewy body disease, motor neuron disease, multiple sclerosis, Parkinson’s disease, Pick’s disease, and other categories, which all revealed significant spatial autocorrelation. The local autocorrelation maps are presented in Figure 7. The types of local clusters differed significantly between diseases. For instance, Lewy body disease seems to split the country into two similar areas, whereas the other disease cluster types appear more dispersed.

##### 3.2.2.2 Municipalities analysis

**Figure 8:**
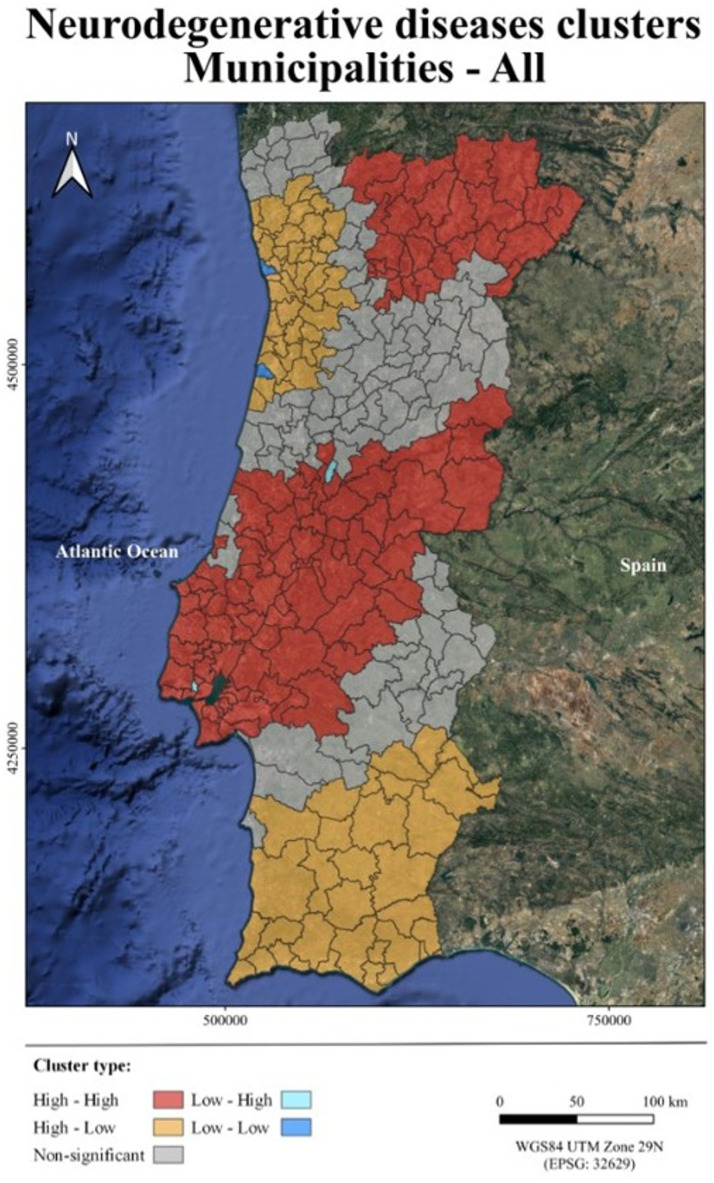
Local neurodegenerative disease clusters when considering municipalities as administrative areas (all diseases). Base map from Google Earth, Map data © 2026 Google. Municipalities / districts data from “DGT - Administrative Limits”, General Directorate of Territory.

The global autocorrelation Moran’s *I* was 0.005 (*p*-value < 0.001) for all diseases, showing a higher spatial autocorrelation than that of the districts. When plotting the local spatial autocorrelation (Figure 8), several clusters appeared: most of the center of Portugal had high-high clusters, while the south had mostly high-low clusters; a couple of isolated low-low clusters appeared in Matosinhos (Porto) and Ílhavo (Aveiro). Also, two low-high clusters were found in Castanheira de Pêra (Leiria) and Amadora (Lisboa).

**Figure 9:**
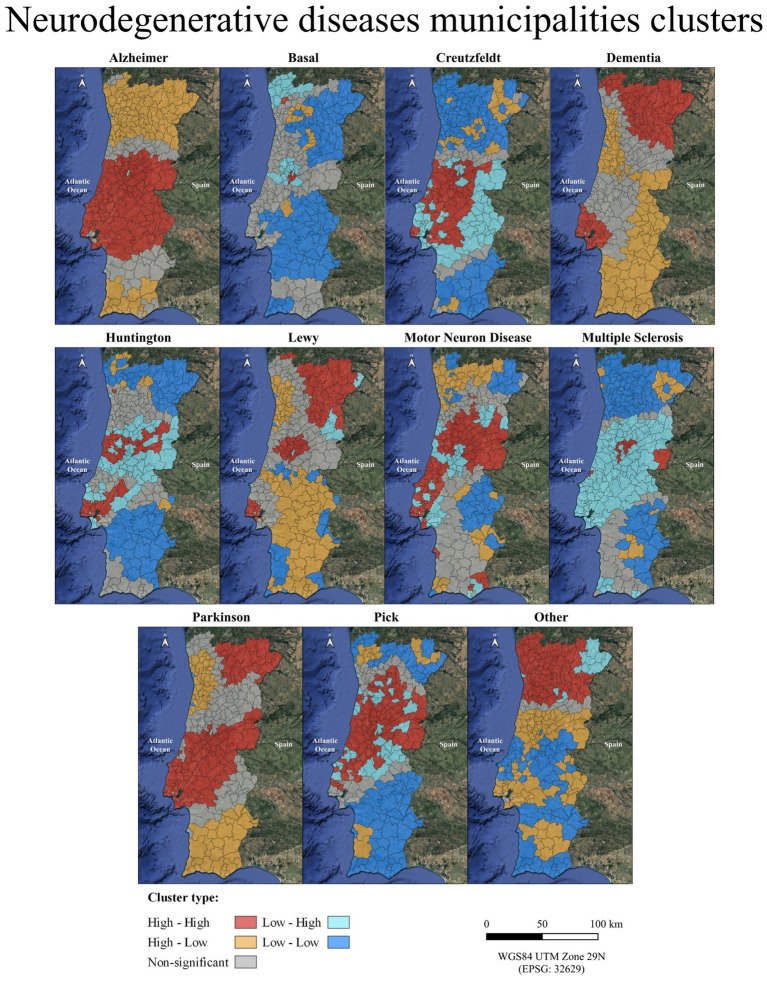
Local neurodegenerative disease clusters when considering municipalities as administrative areas (each disease). Base map from Google Earth, Map data © 2026 Google. Municipalities / districts data from “DGT - Administrative Limits”, General Directorate of Territory.

When examining each disease (Figure 9), some previously observed clusters from the district analysis were split into two different cluster types. For example, multiple sclerosis shows a few municipalities in the northern interior of Portugal that are classified as high-low clusters, where, before, only a low-low cluster was identified in the district. Some clusters also change completely once analyzed at the municipality level, as is the case with Beja in the case of multiple sclerosis, having been non-significant in the districts’ analysis but now showing significantly low clusters throughout most of the district’s municipalities.

## 4 Discussion

This study builds on a previously published preliminary study (26) and is, to the best of the author’s knowledge, the first population-based spatio-temporal descriptive study of neurodegenerative pathologies in Portugal. The dataset, comprising 17 years of hospitalization data from public Portuguese hospitals, provides a substantial sample of neurodegenerative diseases in Portugal, which includes information on patients’ residence at the parish level, allowing these diseases to be analyzed spatially.

### 4.1 Descriptive statistics

Consistent with the literature, the most prevalent age group was patients over 70 years of age, representing 81.3% of our sample (27). The median age was <70 years for Creutzfeldt–Jakob disease, Huntington’s disease, motor neuron disease, multiple sclerosis, and Pick’s disease. Most of our sample was female (56.3%), which is supported by the literature, given that the dataset was dominated by Alzheimer’s disease and dementia hospitalizations (28). Patients were hospitalized for a median of 8 days.

### 4.2 Spatio-temporal analysis

Temporal analysis clearly showed an increase in the ASHR for neurodegenerative diseases throughout all districts. The most affected district was Bragança. An urban–rural dimension may also contribute to these patterns, as metropolitan areas may concentrate hospitalizations because of greater hospital availability and referral flows, whereas less-densely populated interior areas may reflect older population structures or different access to care. However, because this study did not include a direct measure of urbanicity or rurality, these interpretations should be considered cautiously. These findings are broadly consistent with studies from other countries showing that neurodegenerative disease burden is concentrated in older age groups (27) and displays marked geographic variability (11, 29, 30). In this sense, our results add a spatial perspective to Portugal by showing that this variability is more evident at the municipal level than at the district level.

The spatial analysis showed a significantly positive global spatial autocorrelation between both districts and municipalities for all diseases except Alzheimer’s disease and dementia (only when looking at the district data). This correlation is usually higher in municipalities than in districts, suggesting that finer spatial resolution captures greater geographic heterogeneity in hospitalization patterns. While such patterns may be compatible with the influence of localized environmental, socioeconomic, or healthcare-related factors, the present study did not include direct measures of these variables; therefore, causal interpretations should be made with caution. Given the nature of the data, it is more plausible that the observed clustering reflects differences in hospitalization practices, access to care, or disease severity leading to admission rather than disease incidence alone.

The spatial heterogeneity identified in this study should be interpreted in relation to the literature on environmental exposure and pollution. Previous studies have suggested that air pollution may be associated with neurodegenerative outcomes (3), particularly dementia (29) and Parkinson’s disease (30), which were among the most frequent conditions in our dataset. Although the present analysis does not include direct environmental measures, these findings provide a relevant context for interpreting the geographic variability observed here. Nevertheless, any environmental explanation remains speculative and should be tested in future studies using exposure data.

### 4.3 Limitations

The major limitation of our study was the lack of a unique patient identifier or variables to estimate it in our dataset. Therefore, a patient being hospitalized recurrently is treated as independent cases for each hospitalization, overrepresenting diseases that would require repeated hospitalizations. This limitation has direct implications for the interpretation of disease burden and spatial clustering, as areas with higher readmission rates may appear to have disproportionately higher hospitalization levels. To solve this problem, a unique patient identifier would be ideal, and the implementation of other variables, such as birth date and postcode, would also help provide sufficient information to detect duplicate entries.

In addition, the increase in data availability and, consequently, data coding raises awareness that the data are not always comparable throughout larger time periods (31), as is the case with this work. The specification of spatial weights constitutes an important methodological choice. In this study, a fixed 150 km distance band was adopted to ensure full spatial connectivity across mainland Portugal; however, alternative specifications, such as different distance thresholds or contiguity-based weight matrices, may yield variations in the strength or extent of detected spatial clustering. Although the use of a single distance band is common in large-scale descriptive analyses, the absence of formal sensitivity analysis is acknowledged as a limitation. Future studies should assess the robustness of these patterns using multiple spatial weight definitions, particularly hypothesis-driven or confirmatory analyses.

Nevertheless, this is the best available option to study a large sample of neurodegenerative disease patients over the longest available time frame without the implications of collecting the data prospectively. This is only possible through the use of secondary data, which reiterates its importance in the development of new research topics.

### 4.4 Implications and future work

The insights provided by this study on the spatial distribution and characteristics of neurodegenerative diseases in Portugal may be of relevance for any future work that relies on this topic or uses Portuguese public hospitalization data. For instance, future studies on environmental variables may be able to correlate these patterns with new environmental risk factors. In addition, this research provides insights for public health planning and resource allocation, as well as the identification of intervention opportunities to improve patient outcomes. Public health professionals and policymakers should use these findings to enhance awareness of the distribution and impact of neurodegenerative diseases.

## 5 Conclusion

This work is a thorough analysis of the spatio-temporal patterns of neurodegenerative diseases’ age-standardized and population-standardized hospitalization rates in mainland Portugal from 2000 to 2016. The most prevalent disease in this setting was dementia, and the least prevalent was basal ganglia disorders.

Temporal analysis showed increasing hospitalization rates over time, consistent with the aging Portuguese population. In the spatial analysis, a significant positive global spatial autocorrelation was found for most diseases between administrative areas.

With this work, we hope to contribute to the better management of neurodegenerative diseases in Portugal. In particular, to enlighten future studies on this topic, exploring possible determinants of the identified spatio-temporal patterns will contribute to the delineation of strategies aiming to mitigate the impact of neurodegenerative diseases through their prevention and improvement of related health services.

## Data Availability

The data analyzed in this study is subject to the following licenses/restrictions: the data analyzed in this study is not publicly available due to privacy and ethical restrictions. Requests to access these datasets should be directed to Central Administration of the Health System, I. P. (ACSS).
