# Supplementary material for: Spatio-temporal patterns of neurodegenerative disease hospitalizations in mainland Portugal
Source: Front Public Health. 2026 Jun 4;14:1767007. doi: 10.3389/fpubh.2026.1767007 (PMC13277314; doi:10.3389/fpubh.2026.1767007)
Supplement: Supplementary file 1 [file Data_Sheet_1.docx]

Supplementary Material

Table S1 - Hospitalizations with more than one neurodegenerative disease - general descriptive (Creutz. is Creutzfeldt, Dement. is Dementia, Hunt. is Huntington, Park. is Parkinson, N is the number of occurrences and IQR is the inter quartile range).

| **Neurodegenerative diseases** | | | | **N** | **%** | **Sex** | | | | **Age** | | **Length of stay** | |
| --- | --- | --- | --- | --- | --- | --- | --- | --- | --- | --- | --- | --- | --- |
|  |  |  |  |  |  | **Male** | | **Female** | |  |  |  |  |
|  |  |  |  |  |  | **N** | **%** | **N** | **%** | **Median** | **IQR** | **Median** | **IQR** |
| Alzheimer | Basal | - | - | 6 | 0.0 | 2 | 33.3 | 4 | 66.7 | 74 | [70, 79] | 8 | [6, 12] |
|  |  | Dement. | - | 3 | 0.0 | 2 | 66.7 | 1 | 33.3 | 73 | [73, 74] | 18 | [12, 93] |
|  | Creutz. | - | - | 13 | 0.0 | 4 | 30.8 | 9 | 69.2 | 80 | [74, 86] | 11 | [5, 21] |
|  |  | Dement. | - | 4 | 0.0 | 2 | 50 | 2 | 50 | 78 | [75, 81] | 25 | [12, 77] |
|  |  | Other | - | 2 | 0.0 | 1 | 50 | 1 | 50 | 81.5 | [79, 84] | 17 | [11, 24] |
|  | Dement. | - | - | 19110 | 37.3 | 7243 | 37.9 | 11867 | 62.1 | 82 | [77, 86] | 8 | [5, 14] |
|  |  | Hunt. | - | 2 | 0.0 | 1 | 50 | 1 | 50 | 74 | [72, 77] | 16 | [12, 19] |
|  |  |  | Other | 1 | 0.0 | 0 | 0 | 1 | 100 | 72 | [72, 72] | 15 | [15, 15] |
|  |  | Lewy | - | 131 | 0.3 | 66 | 50.4 | 65 | 49.6 | 82 | [78, 85] | 9 | [5, 14] |
|  |  |  | Park. | 2 | 0.0 | 0 | 0 | 2 | 100 | 81 | [79, 84] | 12 | [9, 14] |
|  |  |  | Other | 5 | 0.0 | 3 | 60 | 2 | 40 | 80 | [75, 83] | 6 | [5, 8] |
|  |  | MND | Other | 1 | 0.0 | 1 | 100 | 0 | 0 | 82 | [82, 82] | 1 | [1, 1] |
|  |  | MS | - | 6 | 0.0 | 4 | 66.7 | 2 | 33.3 | 80 | [59, 85] | 18 | [13, 33] |
|  |  | Park. | - | 1143 | 2.2 | 535 | 46.8 | 608 | 53.2 | 81 | [76, 85] | 8 | [5, 14] |
|  |  |  | Other | 27 | 0.1 | 13 | 48.1 | 14 | 51.9 | 81 | [79, 86] | 10 | [5, 14] |
|  |  | Pick | - | 5 | 0.0 | 1 | 20 | 4 | 80 | 73 | [71, 76] | 14 | [7, 16] |
|  |  | Other | - | 488 | 1.0 | 182 | 37.3 | 306 | 62.7 | 81 | [75, 85] | 10 | [5, 18] |
|  | Hunt. | - | - | 8 | 0.0 | 2 | 25 | 6 | 75 | 69 | [62, 77] | 9 | [6, 12] |
|  | Lewy | - | - | 71 | 0.1 | 36 | 50.7 | 35 | 49.3 | 80 | [77, 85] | 9 | [5, 16] |
|  | MND | - | - | 12 | 0.0 | 7 | 58.3 | 5 | 41.7 | 72 | [71, 76] | 20 | [10, 42] |
|  |  | Park. | - | 2 | 0.0 | 0 | 0 | 2 | 100 | 68 | [68, 69] | 12 | [10, 13] |
|  | MS | - | - | 19 | 0.0 | 4 | 21.1 | 15 | 78.9 | 78 | [75, 84] | 8 | [3, 14] |
|  |  | Park. | - | 1 | 0.0 | 0 | 0 | 1 | 100 | 85 | [85, 85] | 7 | [7, 7] |
|  | Park. | - | - | 5391 | 10.5 | 2543 | 47.2 | 2848 | 52.8 | 81 | [76, 85] | 8 | [5, 14] |
|  |  | Lewy | - | 12 | 0.0 | 10 | 83.3 | 2 | 16.7 | 84 | [78, 88] | 9 | [6, 12] |
|  |  | Pick | - | 1 | 0.0 | 1 | 100 | 0 | 0 | 67 | [67, 67] | 8 | [8, 8] |
|  |  | Other | - | 35 | 0.1 | 13 | 37.1 | 22 | 62.9 | 80 | [77, 84] | 11 | [5, 19] |
|  | Pick | - | - | 13 | 0.0 | 8 | 61.5 | 5 | 38.5 | 73 | [69, 80] | 17 | [6, 50] |
|  | Other | - | - | 372 | 0.7 | 140 | 37.6 | 232 | 62.4 | 81 | [77, 85] | 9 | [5, 15] |
| Basal | Creutz. | Dement. | Park. | 1 | 0.0 | 1 | 100 | 0 | 0 | 42 | [42, 42] | 50 | [50, 50] |
|  | Dement. | - | - | 94 | 0.2 | 40 | 42.6 | 54 | 57.4 | 74 | [68, 80] | 10 | [6, 19] |
|  |  | Lewy | - | 4 | 0.0 | 4 | 100 | 0 | 0 | 70.5 | [67, 77] | 14 | [6, 23] |
|  |  | Park. | - | 4 | 0.0 | 2 | 50 | 2 | 50 | 76 | [75, 77] | 9 | [5, 16] |
|  |  | Pick | - | 3 | 0.0 | 2 | 66.7 | 1 | 33.3 | 70 | [70, 72] | 63 | [36, 71] |
|  |  | Other | - | 2 | 0.0 | 0 | 0 | 2 | 100 | 63 | [60, 67] | 12 | [11, 14] |
|  | Hunt. | - | - | 1 | 0.0 | 1 | 100 | 0 | 0 | 60 | [60, 60] | 6 | [6, 6] |
|  | Lewy | - | - | 3 | 0.0 | 1 | 33.3 | 2 | 66.7 | 81 | [77, 84] | 8 | [6, 19] |
|  | MND | - | - | 6 | 0.0 | 3 | 50 | 3 | 50 | 69.5 | [52, 80] | 29 | [6, 89] |
|  |  | Park. | - | 2 | 0.0 | 2 | 100 | 0 | 0 | 70 | [65, 76] | 5 | [3, 6] |
|  | MS | - | - | 3 | 0.0 | 2 | 66.7 | 1 | 33.3 | 57 | [48, 58] | 14 | [13, 14] |
|  | Park. | - | - | 57 | 0.1 | 25 | 43.9 | 32 | 56.1 | 75 | [66, 81] | 8 | [4, 16] |
|  |  | Other | - | 1 | 0.0 | 1 | 100 | 0 | 0 | 64 | [64, 64] | 9 | [9, 9] |
|  | Other | - | - | 10 | 0.0 | 4 | 40 | 6 | 60 | 70.5 | [68, 77] | 12 | [8, 19] |
| Creutz. | Dement. | - | - | 277 | 0.5 | 141 | 50.9 | 136 | 49.1 | 67 | [58, 76] | 22 | [10, 50] |
|  |  | Lewy | - | 3 | 0.0 | 2 | 66.7 | 1 | 33.3 | 65 | [64, 66] | 136 | [84, 378] |
|  |  | MS | - | 1 | 0.0 | 0 | 0 | 1 | 100 | 63 | [63, 63] | 29 | [29, 29] |
|  |  | Park. | - | 9 | 0.0 | 5 | 55.6 | 4 | 44.4 | 72 | [64, 80] | 23 | [12, 33] |
|  |  | Other | - | 10 | 0.0 | 7 | 70 | 3 | 30 | 69 | [60, 74] | 26 | [13, 91] |
|  | Lewy | - | - | 5 | 0.0 | 4 | 80 | 1 | 20 | 43 | [40, 80] | 18 | [10, 26] |
|  |  | MS | - | 1 | 0.0 | 0 | 0 | 1 | 100 | 43 | [43, 43] | 31 | [31, 31] |
|  |  | Other | - | 1 | 0.0 | 1 | 100 | 0 | 0 | 34 | [34, 34] | 11 | [11, 11] |
|  | MND | - | - | 2 | 0.0 | 1 | 50 | 1 | 50 | 68 | [67, 69] | 32 | [17, 46] |
|  | MS | - | - | 30 | 0.1 | 5 | 16.7 | 25 | 83.3 | 37 | [35, 50] | 14 | [5, 48] |
|  | Park. | - | - | 33 | 0.1 | 16 | 48.5 | 17 | 51.5 | 75 | [70, 81] | 11 | [6, 21] |
|  | Pick | - | - | 1 | 0.0 | 1 | 100 | 0 | 0 | 35 | [35, 35] | 6 | [6, 6] |
|  | Other | - | - | 75 | 0.1 | 51 | 68 | 24 | 32 | 45 | [39, 61] | 15 | [10, 31] |
| Dement. | Hunt. | - | - | 130 | 0.3 | 61 | 46.9 | 69 | 53.1 | 63 | [50, 72] | 10 | [7, 19] |
|  |  | Park. | - | 1 | 0.0 | 0 | 0 | 1 | 100 | 75 | [75, 75] | 48 | [48, 48] |
|  |  | Pick | - | 2 | 0.0 | 2 | 100 | 0 | 0 | 47 | [47, 47] | 12 | [6, 17] |
|  |  | Other | - | 2 | 0.0 | 1 | 50 | 1 | 50 | 65.5 | [60, 71] | 21 | [15, 27] |
|  | Lewy | - | - | 1965 | 3.8 | 991 | 50.4 | 974 | 49.6 | 79 | [74, 84] | 10 | [5, 17] |
|  |  | MND | - | 3 | 0.0 | 1 | 33.3 | 2 | 66.7 | 74 | [70, 74] | 12 | [11, 27] |
|  |  | MS | - | 1 | 0.0 | 0 | 0 | 1 | 100 | 69 | [69, 69] | 17 | [17, 17] |
|  |  | Park. | - | 162 | 0.3 | 89 | 54.9 | 73 | 45.1 | 81 | [74, 85] | 8 | [4, 15] |
|  |  |  | Other | 5 | 0.0 | 3 | 60 | 2 | 40 | 77 | [72, 83] | 29 | [6, 33] |
|  |  | Pick | - | 5 | 0.0 | 3 | 60 | 2 | 40 | 65 | [57, 76] | 5 | [4, 6] |
|  |  |  | Other | 1 | 0.0 | 1 | 100 | 0 | 0 | 39 | [39, 39] | 172 | [172, 172] |
|  |  | Other | - | 84 | 0.2 | 40 | 47.6 | 44 | 52.4 | 78 | [74, 83] | 13 | [9, 22] |
|  | MND | - | - | 117 | 0.2 | 60 | 51.3 | 57 | 48.7 | 75 | [66, 81] | 9 | [4, 16] |
|  |  | Park. | - | 14 | 0.0 | 5 | 35.7 | 9 | 64.3 | 72 | [67, 78] | 12 | [7, 18] |
|  |  |  | Pick | 1 | 0.0 | 0 | 0 | 1 | 100 | 71 | [71, 71] | 59 | [59, 59] |
|  |  | Pick | - | 22 | 0.0 | 10 | 45.5 | 12 | 54.5 | 64 | [60, 66] | 12 | [6, 23] |
|  |  | Other | - | 7 | 0.0 | 5 | 71.4 | 2 | 28.6 | 74 | [73, 77] | 15 | [9, 18] |
|  | MS | - | - | 149 | 0.3 | 41 | 27.5 | 108 | 72.5 | 62 | [49, 74] | 9 | [5, 16] |
|  |  | Park. | - | 6 | 0.0 | 4 | 66.7 | 2 | 33.3 | 79 | [75, 81] | 9 | [4, 13] |
|  |  | Other | - | 6 | 0.0 | 2 | 33.3 | 4 | 66.7 | 61 | [54, 64] | 11 | [7, 13] |
|  | Park. | - | - | 14460 | 28.2 | 7087 | 49 | 7373 | 51 | 81 | [76, 86] | 8 | [5, 14] |
|  |  | Pick | - | 27 | 0.1 | 15 | 55.6 | 12 | 44.4 | 68 | [61, 76] | 9 | [7, 22] |
|  |  |  | Other | 1 | 0.0 | 1 | 100 | 0 | 0 | 65 | [65, 65] | 8 | [8, 8] |
|  |  | Other | - | 226 | 0.4 | 112 | 49.6 | 114 | 50.4 | 80 | [74, 85] | 10 | [6, 18] |
|  | Pick | - | - | 614 | 1.2 | 323 | 52.6 | 291 | 47.4 | 67 | [60, 76] | 13 | [6, 27] |
|  |  | Other | - | 23 | 0.0 | 13 | 56.5 | 10 | 43.5 | 64 | [59, 70] | 25 | [10, 49] |
|  | Other | - | - | 4131 | 8.1 | 1868 | 45.2 | 2263 | 54.8 | 81 | [74, 86] | 10 | [6, 18] |
| Hunt. | Lewy | - | - | 1 | 0.0 | 0 | 0 | 1 | 100 | 58 | [58, 58] | 48 | [48, 48] |
|  | MS | - | - | 4 | 0.0 | 0 | 0 | 4 | 100 | 35 | [34, 36] | 12 | [8, 14] |
|  | Park. | - | - | 13 | 0.0 | 5 | 38.5 | 8 | 61.5 | 72 | [66, 79] | 13 | [6, 15] |
|  | Other | - | - | 15 | 0.0 | 11 | 73.3 | 4 | 26.7 | 60 | [47, 74] | 12 | [8, 29] |
| Lewy | MND | - | - | 4 | 0.0 | 0 | 0 | 4 | 100 | 72 | [67, 77] | 7 | [6, 8] |
|  |  | Park. | - | 2 | 0.0 | 2 | 100 | 0 | 0 | 72.5 | [72, 73] | 20 | [14, 25] |
|  | MS | - | - | 15 | 0.0 | 5 | 33.3 | 10 | 66.7 | 43 | [34, 49] | 5 | [4, 11] |
|  |  | Other | - | 3 | 0.0 | 1 | 33.3 | 2 | 66.7 | 50 | [49, 58] | 11 | [9, 11] |
|  | Park. | - | - | 391 | 0.8 | 220 | 56.3 | 171 | 43.7 | 78 | [70, 83] | 9 | [5, 16] |
|  |  | Other | - | 7 | 0.0 | 4 | 57.1 | 3 | 42.9 | 65 | [62, 78] | 17 | [11, 20] |
|  | Pick | - | - | 4 | 0.0 | 2 | 50 | 2 | 50 | 76.5 | [68, 83] | 28 | [13, 56] |
|  | Other | - | - | 53 | 0.1 | 27 | 50.9 | 26 | 49.1 | 74 | [66, 81] | 11 | [6, 22] |
| MND | MS | - | - | 31 | 0.1 | 1 | 3.2 | 30 | 96.8 | 42 | [40, 43] | 4 | [3, 5] |
|  | Park. | - | - | 83 | 0.2 | 41 | 49.4 | 42 | 50.6 | 72 | [68, 79] | 9 | [5, 16] |
|  |  | Pick | - | 1 | 0.0 | 1 | 100 | 0 | 0 | 49 | [49, 49] | 2 | [2, 2] |
|  |  | Other | - | 1 | 0.0 | 0 | 0 | 1 | 100 | 91 | [91, 91] | 26 | [26, 26] |
|  | Pick | - | - | 26 | 0.1 | 15 | 57.7 | 11 | 42.3 | 62.5 | [57, 68] | 6 | [4, 17] |
|  |  | Other | - | 1 | 0.0 | 0 | 0 | 1 | 100 | 56 | [56, 56] | 1 | [1, 1] |
|  | Other | - | - | 41 | 0.1 | 22 | 53.7 | 19 | 46.3 | 71 | [66, 78] | 13 | [7, 21] |
| MS | Park. | - | - | 53 | 0.1 | 23 | 43.4 | 30 | 56.6 | 67 | [62, 77] | 10 | [4, 15] |
|  | Pick | - | - | 2 | 0.0 | 0 | 0 | 2 | 100 | 83 | [83, 83] | 8 | [8, 9] |
|  | Other | - | - | 45 | 0.1 | 21 | 46.7 | 24 | 53.3 | 47 | [38, 55] | 12 | [6, 17] |
| Park. | Pick | - | - | 36 | 0.1 | 23 | 63.9 | 13 | 36.1 | 70 | [63, 76] | 10 | [6, 19] |
|  |  | Other | - | 1 | 0.0 | 1 | 100 | 0 | 0 | 72 | [72, 72] | 12 | [12, 12] |
|  | Other | - | - | 674 | 1.3 | 345 | 51.2 | 329 | 48.8 | 77 | [71, 83] | 9 | [5, 17] |
| Pick | Other | - | - | 23 | 0.0 | 12 | 52.2 | 11 | 47.8 | 68 | [62, 75] | 9 | [5, 22] |

Table S2 - Descriptive statistics of hospitalizations with neurodegenerative disease as primary diagnosis included in this study. Categorical data (sex, age group and residence) is described by its absolute frequency and percentage, while continuous data (age and length of stay, which are both asymmetrically distributed), is described by its median and interquartile range. The total row describes the total frequency of each disease and their respective percentage. yo: years old.

|  |  | **Alzheimer** | **Basal** | **Creutzfeldt** | **Dementia** | **Huntington** | **Lewy** | **Motor Neuron** | **Parkinson** | **Pick** | **Other** | **Total** |
| --- | --- | --- | --- | --- | --- | --- | --- | --- | --- | --- | --- | --- |
| **Sex** | **Female** | 2229 (60.1) | 259 (51.0) | 297 (43.8) | 9225 (54.8) | 238 (51.9) | 624 (52.8) | 1852 (48.2) | 2832 (45.5) | 324 (49.8) | 93 (56.0) | 17973 (52.5) |
|  | **Male** | 1478 (39.9) | 249 (49.0) | 381 (56.2) | 7609 (45.2) | 221 (48.1) | 557 (47.2) | 1994 (51.8) | 3388 (54.5) | 327 (50.2) | 73 (44.0) | 16277 (47.5) |
| **Age (yo)** | | 77 [70, 82] | 65 [55, 72] | 58 [38, 71] | 76 [69, 82] | 50 [41, 60] | 71 [62, 78] | 65 [55, 73] | 68 [60, 76] | 65 [58, 72] | 68 [54, 77] | 73 [63, 80] |
| **Age Group** | **<20 yo** | 3 (0.1) | 26 (5.1) | 67 (9.9) | 28 (0.2) | 15 (3.3) | 53 (4.5) | 244 (6.3) | 21 (0.3) | 1 (0.2) | 10 (6.0) | 468 (1.4) |
|  | **20-70 yo** | 940 (25.4) | 323 (63.6) | 441 (65.0) | 5008 (29.7) | 396 (86.3) | 504 (42.7) | 2359 (61.3) | 3662 (58.9) | 457 (70.2) | 83 (50.0) | 14173 (41.4) |
|  | **>70 yo** | 2764 (74.6) | 159 (31.3) | 170 (25.1) | 11798 (70.1) | 48 (10.5) | 624 (52.8) | 1243 (32.3) | 2537 (40.8) | 193 (29.6) | 73 (44.0) | 19609 (57.3) |
| **Length of stay (days)** | | 7 [3, 16] | 9 [4, 15] | 19 [7, 45] | 10 [4, 20] | 10 [5, 18] | 9 [4, 19] | 6 [3, 13] | 7 [4, 13] | 16 [6, 31] | 8 [2, 14] | 9 [4, 18] |
| **Residence** | **Norte** | 1202 (32.4) | 167 (32.9) | 213 (31.4) | 5815 (34.5) | 105 (22.9) | 385 (32.6) | 1221 (31.7) | 1928 (31.0) | 207 (31.8) | 57 (34.3) | 11300 (33.0) |
|  | **Centro** | 2028 (54.7) | 274 (53.9) | 306 (45.1) | 8710 (51.7) | 279 (60.8) | 679 (57.5) | 1968 (51.2) | 3394 (54.6) | 383 (58.8) | 67 (40.4) | 18088 (52.8) |
|  | **Sul** | 477 (12.9) | 67 (13.2) | 159 (23.5) | 2309 (13.7) | 75 (16.3) | 117 (9.9) | 657 (17.1) | 898 (14.4) | 61 (9.4) | 42 (25.3) | 4862 (14.2) |
| **Total** | | 3707 (10.8) | 508 (1.5) | 678 (2.0) | 16834 (49.2) | 459 (1.3) | 1181 (3.4) | 3846 (11.2) | 6220 (18.2) | 651 (1.9) | 166 (0.5) | 34250 (100) |
